# Tertiary lymphoid organs in systemic autoimmune diseases:  pathogenic or protective?

**DOI:** 10.12688/f1000research.10595.1

**Published:** 2017-02-28

**Authors:** William D. Shipman, Dragos C. Dasoveanu, Theresa T. Lu

**Affiliations:** 1 Autoimmunity and Inflammation Program, Hospital for Special Surgery, New York, NY, USA; 2 Immunology and Microbial Pathogenesis Program, Weill Cornell Graduate School of Medical Sciences, New York, NY, USA; 3 Weill Cornell/Rockefeller/Sloan-Kettering Tri-Institutional MD-PhD Program, New York, NY, USA; 4 Physiology, Biophysics, and Systems Biology Program, Weill Cornell Graduate School of Medical Sciences, New York, NY, USA; 5 Pediatric Rheumatology, Hospital for Special Surgery, New York, NY, USA; 6 Department of Microbiology and Immunology, Weill Cornell Medical College, New York, NY, USA

**Keywords:** Tertiary Lymphoid Organs, Lymph node, Autoimmune disease, Lupus, Rheumatoid Arthritis

## Abstract

Tertiary lymphoid organs are found at sites of chronic inflammation in autoimmune diseases such as systemic lupus erythematosus and rheumatoid arthritis. These organized accumulations of T and B cells resemble secondary lymphoid organs and generate autoreactive effector cells. However, whether they contribute to disease pathogenesis or have protective functions is unclear. Here, we discuss how tertiary lymphoid organs can generate potentially pathogenic cells but may also limit the extent of the response and damage in autoimmune disease.

## Introduction

Autoimmune diseases such as systemic lupus erythematosus (SLE) and rheumatoid arthritis are marked by chronic inflammation in end organs that can be associated with the development of tertiary lymphoid organs (TLOs) ^1– 6^. TLOs are also known as tertiary lymphoid tissues, ectopic lymphoid follicles, or ectopic lymphoid structures and are accumulations of lymphocytes and stromal cells in an organized structure that occur outside of secondary lymphoid organs (SLOs). TLOs share many features with SLOs, such as the presence of T and B cell compartmentalization into T cell zones and B cell follicles, chemokines that mediate the compartmentalization, antigen-presenting cells, lymphatic sinuses, high endothelial venules, follicular dendritic cells, and fibroblastic reticular cells (FRCs) ^7– 11^. In SLE, inflammation in the kidney interstitial tissue is associated with greater risk for kidney failure ^12^. Up to almost half of patients have well-circumscribed aggregates of B cells, plasma cells, and T cells and a small fraction can have well-organized germinal centers with follicular dendritic cells ^1, 2^. In rheumatoid arthritis, TLOs ranging from B and T cell aggregates to germinal centers are found in the inflamed synovium of about half of biopsied patients and are associated with more severe joint and systemic inflammation ^3– 6, 13^. TLOs are also found in other organs in other autoimmune diseases or models, such as in the salivary and lacrimal glands in Sjögren’s syndrome ^14– 16^, the central nervous system in multiple sclerosis ^7, 17– 22^, the pancreas in diabetes ^23– 25^, the thymus in myasthenia gravis ^26, 27^, and the intestines in inflammatory bowel disease ^28, 29^. While findings in recent years have begun to delineate the mechanisms that regulate the formation of TLOs (recently reviewed in 7– 11), it is unclear whether TLOs provide pathogenic or protective contributions to SLE, rheumatoid arthritis, and other autoimmune diseases. Here we will review evidence that TLOs may generate potentially pathogenic cells but that they may limit the extent of pathogenic cell activity.

## Tertiary lymphoid organs can generate potentially pathogenic cells

In the setting of infections, TLOs have been generally considered to be protective, adopting SLO-like functions and acting as “outposts” of SLOs that are directly positioned at the site of inflammation. TLOs form in the lung of influenza-infected mice ^30– 32^ that can maintain and reactivate memory CD8+ T cells ^33^ and produce plasma cells and antiviral serum immunoglobulins ^31^. Remarkably, although there is a delay in anti-viral immunity development, TLOs are sufficient for protection when the hosts are deficient in SLOs ^33^, underscoring the idea that TLOs can generate effector cells that provide effective host defense. Innate lymphoid cells (ILCs) in the lung are induced after influenza infection and have been shown to maintain lung function, epithelial integrity, and airway remodeling ^34^. Although it is unknown whether TLOs play a role in maintaining or stimulating lung ILCs after influenza infection, ILCs have been shown to be associated with TLOs and have even been associated with decreased disease progression in lung cancer ^35– 37^. It is possible that influenza-induced lung TLOs also provide a suitable environment for ILCs to populate and function in a protective manner. Similar to influenza virus infection, TLOs form with pulmonary *Mycobacterium tuberculosis* (MTB) infection ^38– 40^. Latent tuberculosis is associated with more frequent, well-organized TLOs while TLOs are less frequent and less well-formed in active tuberculosis ^40^, suggesting a protective role for TLOs in controlling disease. The TLOs contribute to the formation of granulomas, which function to promote immunity and limit tissue damage ^41^. Additionally, CXCL13 expression that organizes the B cell follicles serves to recruit CXCR5-expressing T helper (Th) cells into granulomas to activate macrophages that are essential to infection control ^38, 40^. These studies on TLOs in infection models highlight the ability of TLOs to support immune responses that are capable of protecting the host.

Similar to immune responses generated in SLOs, immune responses targeted to self may be harmful to the host. The TLOs in SLE kidneys contain germinal centers that show clonal expansion and somatic hypermutation characteristic of germinal center responses in SLOs ^2^, demonstrating well-developed effector responses. The TLOs correlate strongly with the presence of immune complexes, suggesting that the locally generated antibodies are autoantibodies to renal antigens that can fix complement and thus cause tissue inflammation and damage ^2^. Similarly, the B cell responses associated with TLOs in the rheumatoid arthritis synovium ^42^, the salivary glands in Sjögren’s syndrome ^43^, and other target tissues show autoimmunity ^10^. SLE kidneys and rheumatoid synovium are also characterized by the accumulation of Th17 cells, which can have proinflammatory roles ^44– 48^. While IL-17-expressing cells could help to induce TLO formation, as has been shown in the central nervous system and in neonatal lung ^20, 22, 49^, the TLOs could potentially also help to support Th17 cell maintenance or acquisition of additional proinflammatory properties ^20, 50, 51^. Indeed, B cells are necessary for the accumulation of activated T cells, likely by presenting antigen to the T cells ^52– 54^, and B cells in TLOs may be pathogenic in part by stimulating autoreactive T cells, which then can contribute to the inflammatory milieu in the affected end organs. TLOs in autoimmune diseases, then, can be a source of potentially pathogenic lymphocytes.

## Tertiary lymphoid organs can potentially limit pathogenic responses

Despite the generation of autoreactive and proinflammatory cells, TLOs could also have a protective role by sequestering pathogenic lymphocytes and preventing them from leaving the specific tissue or tissue compartment to cause further damage. For example, in SLE, glomerular damage is unrelated to the extent of interstitial inflammation ^12^, but failure to sequester lymphocytes within the interstitial tissue could potentially result in the migration of lymphocytes to the glomeruli and worsened glomerular damage. Alternatively, in the absence of TLOs, the lymphocytes could enter the circulation to home to and potentially damage additional organs outside the kidneys. That inflammatory cells are able to find alternative niches despite the absence of TLOs is seen in MTB infection, where antigen-specific T cells still accumulate, showing an altered, perivascular location, in the absence of TLOs ^38, 40^. Also, B cell selection in the pancreas is unaltered by follicular disruption of TLO in the pancreas of non-obese diabetic mice ^25^. Interestingly, TLOs within tumors but not at the tumor periphery are correlated with good outcomes in a study of pancreatic carcinoma patients ^55^, raising the possibility that the TLOs at the tumor periphery prevent potential anti-tumor lymphocytes from accessing the tumor parenchyma. The concept that TLOs might have a sequestration function is analogous to the sequestration of lymphocytes within SLOs with the S1P agonist fingolimod, which is used to treat multiple sclerosis ^56^. Fingolimod downregulates SIP receptor 1, preventing lymphocyte egress from the SLOs and subsequent migration to end organs ^57^. Sequestration of potentially pathogenic cells, then, may help limit the extent of disease.

TLOs can also be protective if they provide a microenvironment that generates regulatory or reparative cells that reduce the pathogenicity of inflammatory cells. In the apoE-/- model of atherosclerosis, TLOs that form on the outer aspects of the atherosclerotic vessel wall generate regulatory T cells (Tregs). These TLOs are dependent on lymphotoxin β receptor (LTβR) stimulation of presumably local vascular smooth muscle, and preventing TLO formation by deleting LTβR from smooth muscle cells resulted in more and enlarged plaques ^58^. These results suggested that the TLOs were protective, perhaps by the generation of the Tregs. Here, the TLO stroma may be critical for the generation of regulatory cells. Lymph node FRCs have been implicated in Treg generation by presenting self-antigen on MHCII and by guiding T cells into a tolerance-inducing environment ^59, 60^. FRCs, along with endothelial cells, can additionally promote tolerance by MHCI presentation of autoantigen ^61, 62^ and regulate the magnitude of T cell activation by expressing inducible nitric oxide synthase ^63– 65^. Tregs have also been shown to mediate tissue repair via amphiregulin in lung with influenza infection ^66^ and in muscle after injury ^67^, and thus TLO generation of Tregs can have protective effects independent of their immunosuppressive functions. Similarly, ILCs are another source of amphiregulin that is important for repair ^34^, and TLOs may potentially support their development ^68^. Interestingly, both SLE patients and lupus-prone mice possess decreased numbers and abnormal function of Tregs ^69– 72^ while exhibiting increased calcium/calmodulin-dependent protein kinase IV (CamK4) ^73, 74^, which is responsible for an imbalance in Th17 cells and Tregs with a shift towards more Th17 cells. Inhibition of CamK4 corrected this imbalance in lupus-prone mice, decreasing Th17 cells and increasing Tregs in the kidney in association with reduced organ damage ^75^. It is tempting to speculate that the TLOs (and perhaps the SLOs) in SLE do not function correctly to foster optimal Treg generation. TLOs, then, may not only sequester potentially pathogenic cells but also provide an environment that limits the magnitude or severity of the response.

## Conclusion

In conclusion, in autoimmune diseases, TLOs can generate and harbor autoreactive and proinflammatory, potentially pathogenic lymphocytes but could potentially serve to limit pathogenic responses by sequestering these cells or by reducing the magnitude of the response. Therapeutically, targeting TLOs may offer opportunities to ameliorate disease, and more understanding of the potential pathogenic and protective functions is needed. For example, can we identify TLOs that generate more pathogenic cells versus those that have more regulatory functions? Do these different functions in part reflect the evolution of TLO development and maturation? What are the vascular, stromal, and hematopoietic elements that contribute to the different microenvironments, and can we modulate them to generate a more immunoregulatory environment? Furthermore, understanding how the affected tissue outside the TLOs may be similar or distinct in supporting the generation and maintenance of autoreactive lymphocytes would enrich our understanding of the distinct nature of TLOs and also allow us to prevent the lymphocytes from potentially accumulating elsewhere upon TLO disruption. Continued improved understanding of TLO biology will help us better understand how to treat autoimmune disease.

## References

[1] Steinmetz OM Velden J Kneissler U: Analysis and classification of B-cell infiltrates in lupus and ANCA-associated nephritis. *Kidney Int.* 2008;74(4):448–57. 10.1038/ki.2008.191 18528326

[2] Chang A Henderson SG Brandt D: *In situ* B cell-mediated immune responses and tubulointerstitial inflammation in human lupus nephritis. *J Immunol.* 2011;186(3):1849–60. 10.4049/jimmunol.1001983 21187439 PMC3124090

[3] Humby F Bombardieri M Manzo A: Ectopic lymphoid structures support ongoing production of class-switched autoantibodies in rheumatoid synovium. *PLoS Med.* 2009;6(1):e1. 10.1371/journal.pmed.0060001 19143467 PMC2621263

[4] Wengner AM Höpken UE Petrow PK: CXCR5- and CCR7-dependent lymphoid neogenesis in a murine model of chronic antigen-induced arthritis. *Arthritis Rheum.* 2007;56(10):3271–83. 10.1002/art.22939 17907173

[5] Shi K Hayashida K Kaneko M: Lymphoid chemokine B cell-attracting chemokine-1 (CXCL13) is expressed in germinal center of ectopic lymphoid follicles within the synovium of chronic arthritis patients. *J Immunol.* 2001;166(1):650–5. 10.4049/jimmunol.166.1.650 11123349

[6] Takemura S Braun A Crowson C: Lymphoid neogenesis in rheumatoid synovitis. *J Immunol.* 2001;167(2):1072–80. 10.4049/jimmunol.167.2.1072 11441118

[7] Pikor NB Prat A Bar-Or A: Meningeal Tertiary Lymphoid Tissues and Multiple Sclerosis: A Gathering Place for Diverse Types of Immune Cells during CNS Autoimmunity. *Front Immunol.* 2015;6:657. 10.3389/fimmu.2015.00657 26793195 PMC4710700

[8] Barone F Gardner DH Nayar S: Stromal Fibroblasts in Tertiary Lymphoid Structures: A Novel Target in Chronic Inflammation. *Front Immunol.* 2016;7:477. 10.3389/fimmu.2016.00477 27877173 PMC5100680

[9] Buckley CD Barone F Nayar S: Stromal cells in chronic inflammation and tertiary lymphoid organ formation. *Annu Rev Immunol.* 2015;33:715–45. 10.1146/annurev-immunol-032713-120252 25861980

[10] Corsiero E Nerviani A Bombardieri M: Ectopic Lymphoid Structures: Powerhouse of Autoimmunity. *Front Immunol.* 2016;7:430. 10.3389/fimmu.2016.00430 27799933 PMC5066320

[11] Ruddle NH: High Endothelial Venules and Lymphatic Vessels in Tertiary Lymphoid Organs: Characteristics, Functions, and Regulation. *Front Immunol.* 2016;7:491. 10.3389/fimmu.2016.00491 27881983 PMC5101196

[12] Hsieh C Chang A Brandt D: Predicting outcomes of lupus nephritis with tubulointerstitial inflammation and scarring. *Arthritis Care Res (Hoboken).* 2011;63(6):865–74. 10.1002/acr.20441 21309006 PMC3106120

[13] Thurlings RM Wijbrandts CA Mebius RE: Synovial lymphoid neogenesis does not define a specific clinical rheumatoid arthritis phenotype. *Arthritis Rheum.* 2008;58(6):1582–9. 10.1002/art.23505 18512774

[14] Fava RA Kennedy SM Wood SG: Lymphotoxin-beta receptor blockade reduces CXCL13 in lacrimal glands and improves corneal integrity in the NOD model of Sjögren's syndrome. *Arthritis Res Ther.* 2011;13(6):R182. 10.1186/ar3507 22044682 PMC3334628

[15] Bombardieri M Barone F Lucchesi D: Inducible tertiary lymphoid structures, autoimmunity, and exocrine dysfunction in a novel model of salivary gland inflammation in C57BL/6 mice. *J Immunol.* 2012;189(7):3767–76. 10.4049/jimmunol.1201216 22942425 PMC3448973

[16] Holdgate N St Clair EW: Recent advances in primary Sjogren's syndrome [version 1; referees: 3 approved]. *F1000Res.* 2016;5: pii: F1000 Faculty Rev-1412. 10.12688/f1000research.8352.1 27347394 PMC4916986

[17] Columba-Cabezas S Griguoli M Rosicarelli B: Suppression of established experimental autoimmune encephalomyelitis and formation of meningeal lymphoid follicles by lymphotoxin beta receptor-Ig fusion protein. *J Neuroimmunol.* 2006;179(1–2):76–86. 10.1016/j.jneuroim.2006.06.015 16870269

[18] Magliozzi R Columba-Cabezas S Serafini B: Intracerebral expression of CXCL13 and BAFF is accompanied by formation of lymphoid follicle-like structures in the meninges of mice with relapsing experimental autoimmune encephalomyelitis. *J Neuroimmunol.* 2004;148(1–2):11–23. 10.1016/j.jneuroim.2003.10.056 14975582

[19] Mitsdoerffer M Peters A: Tertiary Lymphoid Organs in Central Nervous System Autoimmunity. *Front Immunol.* 2016;7:451. 10.3389/fimmu.2016.00451 27826298 PMC5078318

[20] Peters A Pitcher LA Sullivan JM: Th17 cells induce ectopic lymphoid follicles in central nervous system tissue inflammation. *Immunity.* 2011;35(6):986–96. 10.1016/j.immuni.2011.10.015 22177922 PMC3422678

[21] Serafini B Rosicarelli B Magliozzi R: Detection of ectopic B-cell follicles with germinal centers in the meninges of patients with secondary progressive multiple sclerosis. *Brain Pathol.* 2004;14(2):164–74. 10.1111/j.1750-3639.2004.tb00049.x 15193029 PMC8095922

[22] Pikor NB Astarita JL Summers-Deluca L: Integration of Th17- and Lymphotoxin-Derived Signals Initiates Meningeal-Resident Stromal Cell Remodeling to Propagate Neuroinflammation. *Immunity.* 2015;43(6):1160–73. 10.1016/j.immuni.2015.11.010 26682987

[23] Astorri E Bombardieri M Gabba S: Evolution of ectopic lymphoid neogenesis and *in situ* autoantibody production in autoimmune nonobese diabetic mice: cellular and molecular characterization of tertiary lymphoid structures in pancreatic islets. *J Immunol.* 2010;185(6):3359–68. 10.4049/jimmunol.1001836 20713891

[24] Ludewig B Odermatt B Landmann S: Dendritic cells induce autoimmune diabetes and maintain disease via *de novo* formation of local lymphoid tissue. *J Exp Med.* 1998;188(8):1493–501. 10.1084/jem.188.8.1493 9782126 PMC2213416

[25] Henry RA Kendall PL: CXCL13 blockade disrupts B lymphocyte organization in tertiary lymphoid structures without altering B cell receptor bias or preventing diabetes in nonobese diabetic mice. *J Immunol.* 2010;185(3):1460–5. 10.4049/jimmunol.0903710 20574003 PMC3824617

[26] Hill ME Shiono H Newsom-Davis J: The myasthenia gravis thymus: a rare source of human autoantibody-secreting plasma cells for testing potential therapeutics. *J Neuroimmunol.* 2008;201–202:50–6. 10.1016/j.jneuroim.2008.06.027 18722675

[27] Zhang X Liu S Chang T: Intrathymic Tfh/B Cells Interaction Leads to Ectopic GCs Formation and Anti-AChR Antibody Production: Central Role in Triggering MG Occurrence. *Mol Neurobiol.* 2016;53(1):120–31. 10.1007/s12035-014-8985-1 25407929

[28] Buettner M Lochner M: Development and Function of Secondary and Tertiary Lymphoid Organs in the Small Intestine and the Colon. *Front Immunol.* 2016;7:342. 10.3389/fimmu.2016.00342 27656182 PMC5011757

[29] Olivier BJ Cailotto C van der Vliet J: Vagal innervation is required for the formation of tertiary lymphoid tissue in colitis. *Eur J Immunol.* 2016;46(10):2467–80. 10.1002/eji.201646370 27457277

[30] Moyron-Quiroz JE Rangel-Moreno J Kusser K: Role of inducible bronchus associated lymphoid tissue (iBALT) in respiratory immunity. *Nat Med.* 2004;10(9):927–34. 10.1038/nm1091 15311275

[31] GeurtsvanKessel CH Willart MA Bergen IM: Dendritic cells are crucial for maintenance of tertiary lymphoid structures in the lung of influenza virus-infected mice. *J Exp Med.* 2009;206(11):2339–49. 10.1084/jem.20090410 19808255 PMC2768850

[32] Halle S Dujardin HC Bakocevic N: Induced bronchus-associated lymphoid tissue serves as a general priming site for T cells and is maintained by dendritic cells. *J Exp Med.* 2009;206(12):2593–601. 10.1084/jem.20091472 19917776 PMC2806625

[33] Moyron-Quiroz JE Rangel-Moreno J Hartson L: Persistence and responsiveness of immunologic memory in the absence of secondary lymphoid organs. *Immunity.* 2006;25(4):643–54. 10.1016/j.immuni.2006.08.022 17045819

[34] Monticelli LA Sonnenberg GF Abt MC: Innate lymphoid cells promote lung-tissue homeostasis after infection with influenza virus. *Nat Immunol.* 2011;12(11):1045–54. 21946417 10.1031/ni.2131 PMC3320042

[35] Carrega P Loiacono F Di Carlo E: NCR ^+^ILC3 concentrate in human lung cancer and associate with intratumoral lymphoid structures. *Nat Commun.* 2015;6:8280. 10.1038/ncomms9280 26395069

[36] Meier D Bornmann C Chappaz S: Ectopic lymphoid-organ development occurs through interleukin 7-mediated enhanced survival of lymphoid-tissue-inducer cells. *Immunity.* 2007;26(5):643–54. 10.1016/j.immuni.2007.04.009 17521585

[37] Schmutz S Bosco N Chappaz S: Cutting edge: IL-7 regulates the peripheral pool of adult ROR gamma ^+^ lymphoid tissue inducer cells. *J Immunol.* 2009;183(4):2217–21. 10.4049/jimmunol.0802911 19635901

[38] Khader SA Rangel-Moreno J Fountain JJ: In a murine tuberculosis model, the absence of homeostatic chemokines delays granuloma formation and protective immunity. *J Immunol.* 2009;183(12):8004–14. 10.4049/jimmunol.0901937 19933855 PMC2799945

[39] Ulrichs T Kosmiadi GA Trusov V: Human tuberculous granulomas induce peripheral lymphoid follicle-like structures to orchestrate local host defence in the lung. *J Pathol.* 2004;204(2):217–28. 10.1002/path.1628 15376257

[40] Slight SR Rangel-Moreno J Gopal R: CXCR5 ^+^ T helper cells mediate protective immunity against tuberculosis. *J Clin Invest.* 2013;123(2):712–26. 10.1172/JCI65728 23281399 PMC3561804

[41] Bean AG Roach DR Briscoe H: Structural deficiencies in granuloma formation in TNF gene-targeted mice underlie the heightened susceptibility to aerosol *Mycobacterium tuberculosis* infection, which is not compensated for by lymphotoxin. *J Immunol.* 1999;162(6):3504–11. 10092807

[42] Corsiero E Bombardieri M Carlotti E: Single cell cloning and recombinant monoclonal antibodies generation from RA synovial B cells reveal frequent targeting of citrullinated histones of NETs. *Ann Rheum Dis.* 2016;75(10):1866–75. 10.1136/annrheumdis-2015-208356 26659717 PMC5036240

[43] Salomonsson S Jonsson MV Skarstein K: Cellular basis of ectopic germinal center formation and autoantibody production in the target organ of patients with Sjögren's syndrome. *Arthritis Rheum.* 2003;48(11):3187–201. 10.1002/art.11311 14613282

[44] Pisitkun P Ha HL Wang H: Interleukin-17 cytokines are critical in development of fatal lupus glomerulonephritis. *Immunity.* 2012;37(6):1104–15. 10.1016/j.immuni.2012.08.014 23123062 PMC3594848

[45] Zhang Z Kyttaris VC Tsokos GC: The role of IL-23/IL-17 axis in lupus nephritis. *J Immunol.* 2009;183(5):3160–9. 10.4049/jimmunol.0900385 19657089 PMC2766304

[46] Crispín JC Oukka M Bayliss G: Expanded double negative T cells in patients with systemic lupus erythematosus produce IL-17 and infiltrate the kidneys. *J Immunol.* 2008;181(12):8761–6. 10.4049/jimmunol.181.12.8761 19050297 PMC2596652

[47] Genovese MC Durez P Richards HB: One-year efficacy and safety results of secukinumab in patients with rheumatoid arthritis: phase II, dose-finding, double-blind, randomized, placebo-controlled study. *J Rheumatol.* 2014;41(3):414–21. 10.3899/jrheum.130637 24429175

[48] Hirota K Yoshitomi H Hashimoto M: Preferential recruitment of CCR6-expressing Th17 cells to inflamed joints via CCL20 in rheumatoid arthritis and its animal model. *J Exp Med.* 2007;204(12):2803–12. 10.1084/jem.20071397 18025126 PMC2118525

[49] Rangel-Moreno J Carragher DM de la Luz Garcia-Hernandez M: The development of inducible bronchus-associated lymphoid tissue depends on IL-17. *Nat Immunol.* 2011;12(7):639–46. 10.1038/ni.2053 21666689 PMC3520063

[50] Nistala K Adams S Cambrook H: Th17 plasticity in human autoimmune arthritis is driven by the inflammatory environment. *Proc Natl Acad Sci U S A.* 2010;107(33):14751–6. 10.1073/pnas.1003852107 20679229 PMC2930428

[51] Harbour SN Maynard CL Zindl CL: Th17 cells give rise to Th1 cells that are required for the pathogenesis of colitis. *Proc Natl Acad Sci U S A.* 2015;112(22):7061–6. 10.1073/pnas.1415675112 26038559 PMC4460486

[52] Chan OT Madaio MP Shlomchik MJ: B cells are required for lupus nephritis in the polygenic, Fas-intact MRL model of systemic autoimmunity. *J Immunol.* 1999;163(7):3592–6. 10490951

[53] Chan OT Hannum LG Haberman AM: A novel mouse with B cells but lacking serum antibody reveals an antibody-independent role for B cells in murine lupus. *J Exp Med.* 1999;189(10):1639–48. 10.1084/jem.189.10.1639 10330443 PMC2193634

[54] Weyand CM Goronzy JJ Takemura S: Cell-cell interactions in synovitis. Interactions between T cells and B cells in rheumatoid arthritis. *Arthritis Res.* 2000;2(6):457–63. 10.1186/ar128 11094459 PMC128875

[55] Hiraoka N Ino Y Yamazaki-Itoh R: Intratumoral tertiary lymphoid organ is a favourable prognosticator in patients with pancreatic cancer. *Br J Cancer.* 2015;112(11):1782–90. 10.1038/bjc.2015.145 25942397 PMC4647237

[56] Brinkmann V Billich A Baumruker T: Fingolimod (FTY720): discovery and development of an oral drug to treat multiple sclerosis. *Nat Rev Drug Discov.* 2010;9(11):883–97. 10.1038/nrd3248 21031003

[57] Matloubian M Lo CG Cinamon G: Lymphocyte egress from thymus and peripheral lymphoid organs is dependent on S1P receptor 1. *Nature.* 2004;427(6972):355–60. 10.1038/nature02284 14737169

[58] Hu D Mohanta SK Yin C: Artery Tertiary Lymphoid Organs Control Aorta Immunity and Protect against Atherosclerosis via Vascular Smooth Muscle Cell Lymphotoxin β Receptors. *Immunity.* 2015;42(6):1100–15. 10.1016/j.immuni.2015.05.015 26084025 PMC4678289

[59] Baptista AP Roozendaal R Reijmers RM: Lymph node stromal cells constrain immunity via MHC class II self-antigen presentation. *eLife.* 2014;3:e04433. 10.7554/eLife.04433 25407678 PMC4270074

[60] Warren KJ Iwami D Harris DG: Laminins affect T cell trafficking and allograft fate. *J Clin Invest.* 2014;124(5):2204–18. 10.1172/JCI73683 24691446 PMC4001556

[61] Fletcher AL Lukacs-Kornek V Reynoso ED: Lymph node fibroblastic reticular cells directly present peripheral tissue antigen under steady-state and inflammatory conditions. *J Exp Med.* 2010;207(4):689–97. 10.1084/jem.20092642 20308362 PMC2856033

[62] Cohen JN Guidi CJ Tewalt EF: Lymph node-resident lymphatic endothelial cells mediate peripheral tolerance via Aire-independent direct antigen presentation. *J Exp Med.* 2010;207(4):681–8. 10.1084/jem.20092465 20308365 PMC2856027

[63] Lukacs-Kornek V Malhotra D Fletcher AL: Regulated release of nitric oxide by nonhematopoietic stroma controls expansion of the activated T cell pool in lymph nodes. *Nat Immunol.* 2011;12(11):1096–104. 10.1038/ni.2112 21926986 PMC3457791

[64] Siegert S Huang HY Yang CY: Fibroblastic reticular cells from lymph nodes attenuate T cell expansion by producing nitric oxide. *PLoS One.* 2011;6(11):e27618. 10.1371/journal.pone.0027618 22110693 PMC3215737

[65] Khan O Headley M Gerard A: Regulation of T cell priming by lymphoid stroma. *PLoS One.* 2011;6(11):e26138. 10.1371/journal.pone.0026138 22110583 PMC3215700

[66] Arpaia N Green JA Moltedo B: A Distinct Function of Regulatory T Cells in Tissue Protection. *Cell.* 2015;162(5):1078–89. 10.1016/j.cell.2015.08.021 26317471 PMC4603556

[67] Burzyn D Kuswanto W Kolodin D: A special population of regulatory T cells potentiates muscle repair. *Cell.* 2013;155(6):1282–95. 10.1016/j.cell.2013.10.054 24315098 PMC3894749

[68] Barone F Nayar S Campos J: IL-22 regulates lymphoid chemokine production and assembly of tertiary lymphoid organs. *Proc Natl Acad Sci U S A.* 2015;112(35):11024–9. 10.1073/pnas.1503315112 26286991 PMC4568258

[69] Valencia X Yarboro C Illei G: Deficient CD4 ^+^CD25 ^high^ T regulatory cell function in patients with active systemic lupus erythematosus. *J Immunol.* 2007;178(4):2579–88. 10.4049/jimmunol.178.4.2579 17277168

[70] Hsu W Suen J Chiang B: The role of CD4 ^+^CD25 ^+^ T cells in autoantibody production in murine lupus. *Clin Exp Immunol.* 2006;145(3):513–9. 10.1111/j.1365-2249.2006.03173.x 16907921 PMC1809714

[71] Scalapino KJ Tang Q Bluestone JA: Suppression of disease in New Zealand Black/New Zealand White lupus-prone mice by adoptive transfer of *ex vivo* expanded regulatory T cells. *J Immunol.* 2006;177(3):1451–9. 10.4049/jimmunol.177.3.1451 16849451

[72] Liu MF Wang CR Fung LL: Decreased CD4 ^+^CD25 ^+^ T cells in peripheral blood of patients with systemic lupus erythematosus. *Scand J Immunol.* 2004;59(2):198–202. 10.1111/j.0300-9475.2004.01370.x 14871297

[73] Juang YT Wang Y Solomou EE: Systemic lupus erythematosus serum IgG increases CREM binding to the *IL-2* promoter and suppresses IL-2 production through CaMKIV. *J Clin Invest.* 2005;115(4):996–1005. 10.1172/JCI22854 15841182 PMC1070410

[74] Koga T Ichinose K Mizui M: Calcium/calmodulin-dependent protein kinase IV suppresses IL-2 production and regulatory T cell activity in lupus. *J Immunol.* 2012;189(7):3490–6. 10.4049/jimmunol.1201785 22942433 PMC3448834

[75] Koga T Mizui M Yoshida N: KN-93, an inhibitor of calcium/calmodulin-dependent protein kinase IV, promotes generation and function of Foxp3 ^+^ regulatory T cells in MRL/ *lpr* mice. *Autoimmunity.* 2014;47(7):445–50. 10.3109/08916934.2014.915954 24829059 PMC4341833

